# Identification of a novel splice-site mutation in *MIP* in a Chinese congenital cataract family

**Published:** 2009-01-12

**Authors:** Jin Jiang, Chongfei Jin, Wei Wang, Xiajing Tang, Xingchao Shentu, Renyi Wu, Yao Wang, Kun Xia, Ke Yao

**Affiliations:** 1 Eye Center of the 2nd Affiliated Hospital, Medical College of Zhejiang University, Hangzhou, China; 2 Department of Ophthalmology, Zhejiang Provincial People’s Hospital, Hangzhou, China; 3 National Laboratory of Medical Genetics of China, Changsha, China

## Abstract

**Purpose:**

To map the locus and identify the gene causing autosomal dominant congenital cataract (ADCC) with “snail-like” phenotype in a large Chinese family.

**Methods:**

Clinical and ophthalmologic examinations were conducted on family members and documented by slit lamp photography. Linkage analysis was performed with an initial 41 microsatellite markers, then 3 additional markers flanking the major intrinsic protein (*MIP*) gene. Mutations were screened by DNA sequencing and verified by restriction fragment length polymorphism (RFLP) analysis.

**Results:**

Significant two-point LOD scores were obtained at 5 markers flanking *MIP* with the highest 3.08 (θ=0.00) at marker D12S1632. Mutation screening of *MIP* identified a heterozygous G>A transition at the acceptor splice site of intron 3 (IVS3 −1 G>A), abolishing a BstSF I restriction site in one allele of all the affected individuals.

**Conclusions:**

We identified a novel splice-site mutation (IVS3 −1 G>A in *MIP*) in a Chinese ADCC family. To our knowledge, this is the first report on an acceptor splice-site mutation in human genes associated with ADCC.

## Introduction

Although surgical techniques and visual prognosis have been greatly improved in recent times, congenital cataracts remain the leading cause of visual disability in children worldwide. Without prompt treatment, cataracts can occlude clear imaging on the retina, resulting in failure to develop normal retinal-cortical synaptic connections and finally, irreversible amblyopia. Approximately 50% of congenital cataracts are inherited, with the most common being the autosomal dominant form [1]. To date, more than 25 independent loci and 17 cataract-related genes have been identified as being associated with isolated autosomal dominant congenital cataract (ADCC) [2]. These genes can be divided into 5 groups including: (1) Genes encoding crystallins: *CRYAA*, *CRYAB*, *CRYBA1/A3*, *CRYBA4*, *CRYBB1*, *CRYBB2*, *CRYGC*, *CRYGD*, and *CRYGS* [3-11]; (2) Genes encoding membrane transport and channel proteins: *GJA3*, *GJA8*, and *MIP* (also know as *AQP0*) [12-14]; (3) Genes encoding cytoskeletal proteins such as *BFSP2* [15,16]; (4) Genes encoding transcription factors such as *PITX3* and *HSF4* [17,18]; and (5) Others: *CHMP4B* [19] and *EPHA2* [20]. Most of the mutations detected in these genes are missense and nonsense mutations [21]. Few splice-site mutations have ever been reported associated with ADCC, except in *CRYBA1/A3* and *HSF4* [22-24]. Furthermore, to our knowledge, there is no report on an acceptor splice-site mutation in human genes associated with ADCC.

In this study, we identified *MIP* as the disease-causing gene in a four-generation Chinese family with ADCC by linkage analysis, and detected a novel G>A transition at the acceptor splice site of intron 3 of the *MIP* gene.

## Methods

### Family data and genomic DNA preparation

**Figure 1:**
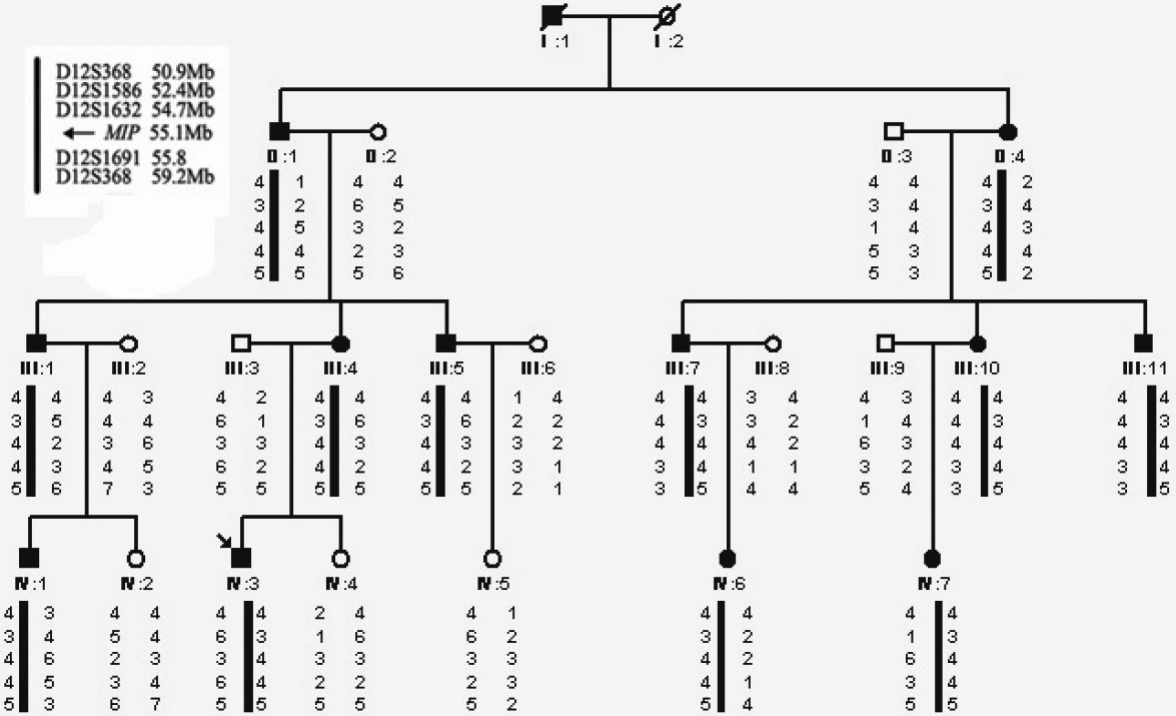
Pedigree of the Chinese cataract family and haplotype analysis. Squares and circles indicate males and females, respectively. Solid and open symbols denote affected and unaffected individuals, respectively. Haplotype analysis shows the segregation of five microsatellite markers on chromosome 12. The physical distance of microsatellite markers and the disease gene have been given in the top left corner. The haplotype of the disease-bearing chromosome is indicated by black bar.

A four-generation family with ADCC was ascertained through the Eye Center of the 2nd Affiliated Hospital, Medical College of Zhejiang University, Hangzhou, China. Appropriate informed consent was obtained from all participants and the study protocol adhered to the principles of the Declaration of Helsinki. Twenty-two individuals (12 affected and 10 unaffected) from the family were enrolled in the study (Figure 1). Affected status was determined by a history of cataract extraction or ophthalmologic examination, including visual acuity, slit lamp, and fundus examination. The phenotypes were documented by slit lamp photography. Blood specimens (5 ml) from all the patients and available family members were collected in a BD Vacutainer^®^ (BD Biosciences, San Jose, CA) containing EDTA. Genomic DNA was isolated as previously described [25]. Mutation nomenclature follows the guidelines of the Human Genome Variation Society (HGV) with the numbering based on +1 as the A of the ATG translation initiation codon in the reference sequence. The initiation codon is codon 1.

### Genotyping and linkage analysis

Genotyping was performed as described previously, using the initial 41 microsatellite markers, corresponding to 18 known candidate loci for ADCC [23,26], and then another 3 markers localized to 12q13. Two-point disease to marker linkage analysis was conducted by the MLINK routine of the LINKAGE software package, version 5.1. The disease locus was specified to be an autosomal dominant trait with a disease allele frequency of 0.0001. The allele frequencies for each marker were assumed to be equal as were the recombination frequencies in males and females. Genetic penetrance was assigned to be full.

### PCR and DNA sequencing

Gene specific PCR primers for *MIP* were designed flanking each exon and intron-exon junction (Table 1). The cycling conditions for PCR were as follows: 95 °C preactivation for 5 min, 10 cycles of touchdown PCR with 0.5 °C down per cycle from 62 °C to 57 °C, followed by 25 cycles with denaturation at 94 °C for 45 s, annealing at 58 °C for 45 s and extension at 72 °C for 45 s. PCR products were isolated by electrophoresis on 3% agarose gels and sequenced using the BigDye Terminator Cycle sequencing kit V 3.1(ABI Applied Biosystems; Sangon Co., Shanghai, China) on an ABI PRISM 3730 Sequence Analyzer (ABI), according to the manufacturer’s directions.

**Table 1:** Primers and product sizes of *MIP*.

**Name**	**Primer**	**Product size (bp)**
exon 1F	5′-GACTGTCCACCCAGACAAGG-3′	492
exon 1R	5′-TCAGGGAGTCAGGGCAATAG-3′	
exon 2F	5′-TGAAGGAGCACTGTTAGGAGATG-3′	500
exon 2R	5′-AGAGGGATAGGGCAGAGTTGATT-3′	
exon 3F	5′-CCAGACAGGGCATCAGT-3′	373
exon 3R	5′-TGGTACAGCAGCCAACAC-3′	
exon 4F	5′-AAGGTGTGGGATAAAGGAGT-3′	429
exon 4R	5′-TTCTTCATCTAGGGGCTGGC-3′	

### Restriction fragment length polymorphism (RFLP) analysis

After identifying an acceptor splice-site mutation in the intron 3-exon 4 junction, all family members and 100 unrelated control individuals were examined by RFLP analysis. The mutation abolished a BstSF I site. PCR products of exon 4 of *MIP* were digested for 1 h at 60 °C with BstSF I (Bio Basic Inc., Markham, Canada) and separated on a 3% agarose gel by electrophoresis.

## Results

### Clinical evaluation

**Figure 2:**
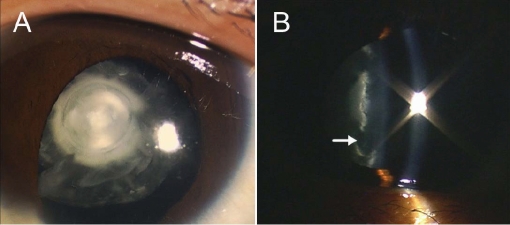
Photographs of the left eye of the proband with congenital cataract. **A:** Diffuse illumination shows a “snail-like” cataract with opacity density gradually increased from the peripheral adult nucleus to the inner embryonal nucleus, while the cortex remains transparent. **B:** Slit section shows that the opacified nuclei are separated by a transparent circle (white arrow).

We identified a four-generation Chinese family with clear diagnosis of ADCC. Opacification of the lens was bilateral in all the affected individuals. Most of the patients had nystagmus with visual acuity ranging from hand move to 15/60 in the unoperated eyes. There was no family history of other ocular or systemic abnormalities. The lens opacification of the proband was very unique giving an appearance of a snail, with opacity density gradually increasing from the peripheral adult nucleus to the inner embryonal nucleus. The cortex remained transparent and the nuclei were separated by a transparent circle (Figure 2).

### Linkage analysis

After the other candidate loci were excluded, positive two-point LOD scores were obtained at markers D12S368 (Zmax=1.66 at θ=0.0) and D12S83 (Zmax=2.01 at θ=0.0). *MIP* was flanked by these two markers. Therefore, three additional markers very near to *MIP* were subsequently used for further confirmation. All of these 5 markers received significant scores, and the maximum score was obtained with marker D12S1632 (Zmax=3.08 at θ=0.0; Table 2).

**Table 2:** Two-point LOD scores for linkage between autosomal dominant congenital cataract locus and chromosome 12 markers.

**Markers and *MIP***	**Physical distance (Mbp)**	**LOD scores by recombination fraction (θ)**					
		**0**	**0.1**	**0.2**	**0.3**	**0.4**	**0.5**
D12S368	50.9177–50.9179	1.66	1.32	0.95	0.56	0.19	0
D12S1586	52.4330–52.4333	2.78	2.18	1.57	0.96	0.42	0
D12S1632	54.7016–54.7019	3.08	2.52	1.91	1.24	0.55	0
*MIP*	55.1300–55.1346						
D12S1691	55.7920–55.7923	2.23	1.85	1.44	1.00	0.53	0
D12S83	59.1756–59.1759	2.01	1.62	1.21	0.77	0.36	0

### Mutation analysis

**Figure 3:**
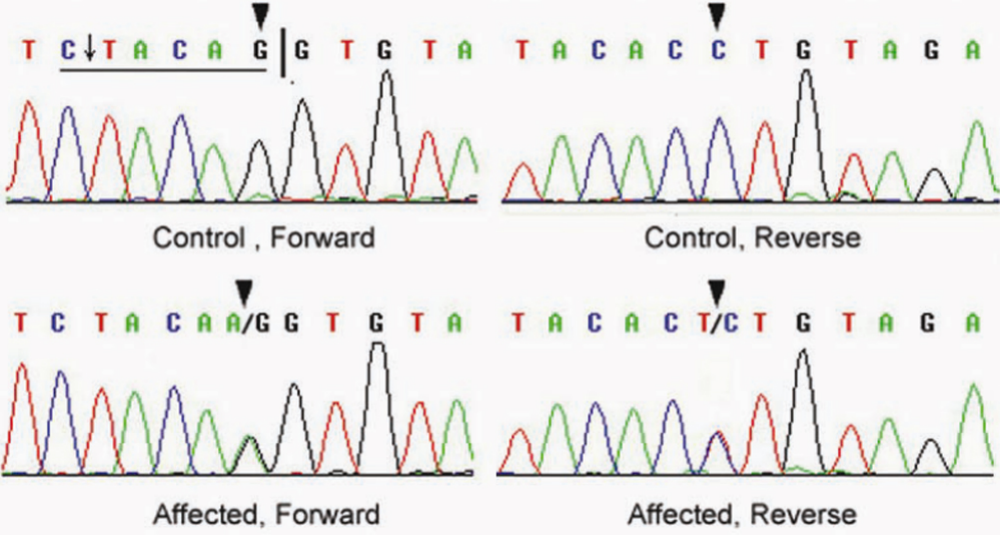
Forward and reverse sequence analysis of the affected and unaffected individuals in this ADCC Chinese family. It shows a heterozygous mutation (IVS3–1 G>A) in the third canonical AG sites of *MIP* (black triangles). The black vertical line denotes the normal intron 3-exon 4 acceptor splice site. The mutation IVS3 −1 G>A abolishes a BstSF I site (underlined) which is enzymatic cut indicated by the arrow.

**Figure 4:**
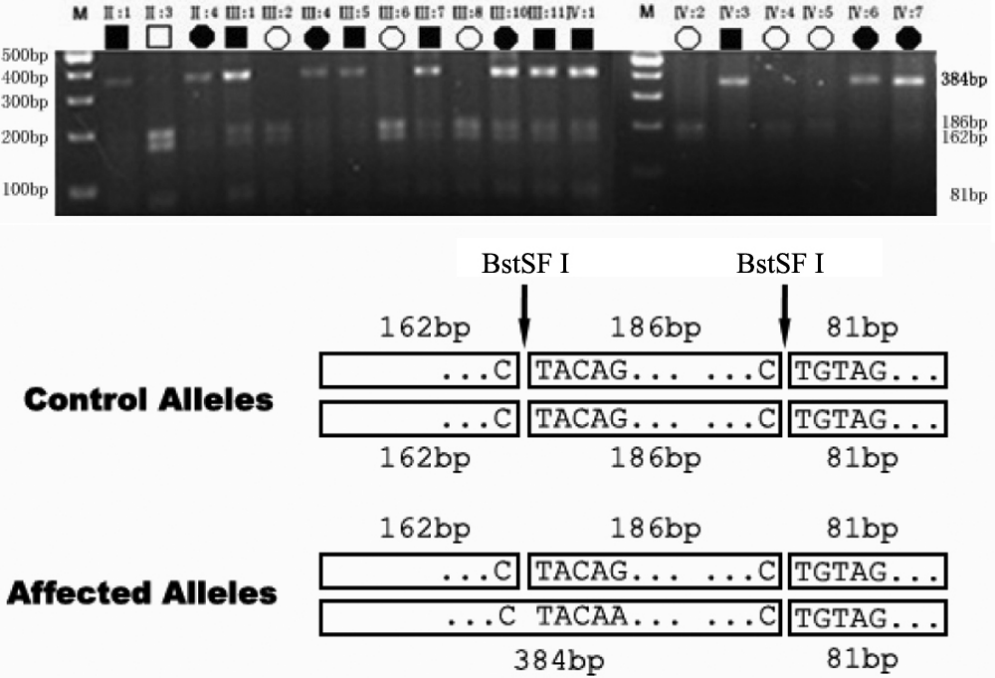
Restriction fragment length polymorphism (RFLP) analysis showing that the abolishment of a BstSF I site cosegregates with affected individuals. The PCR product of exon 4 with flanking sequences includes 429 bp with 2 BstSF I sites (CTACAG and CTGTAG). The unaffected has three fragments (81 bp, 162 bp, and 186 bp) after BstSF I digestion, whereas the affected has four (81 bp, 162 bp, 186 bp, and the crucial 348 bp). Only the affected allele shows the 384bp band. M means DNA ladder marker.

By sequencing the PCR products of *MIP*, we identified a single base substitution in the acceptor splice site of intron 3 (IVS3 −1 G>A) which cosegregated with all affected individuals, whereas this heterozygous mutation was not present in the unaffected family members (Figure 3). The IVS3 −1 G>A mutation changed the canonical 3′ acceptor splice site of intron 3 from AG to AA, resulting in a BstSF I restriction site abolishment. RFLP analysis verified the mutation and showed it cosegregation with all affected individuals. The mutation was not detected in unaffected family members and 100 unrelated Chinese without cataract as control (Figure 4).

## Discussion

In this report, we first identified an acceptor splice-site mutation (IVS3 −1 G>A in *MIP*) associated with ADCC in a four-generation Chinese family. To date, five other mutations in *MIP* have been identified from five unrelated human families (c.413C>G, c.401A>G, c.638delG, c.97C>T, and c.702G>A). Individuals with c.413C>G, c.638delG, and c.702G>A mutations have polymorphic cataracts [14,27-29]. The c.401A>G mutation causes nonprogressive lamellar cataract with sutural opacities and c.97C>T mutation causes total cataract [14,29]. In those affected with the IVS3 −1 G>A mutation, a unique cataract phenotype was observed. The proband demonstrates a “snail-like” cataract with opacity density gradually increasing from the peripheral adult nucleus to the inner embryonal nucleus, while the cortex remains transparent. It indicates that the dysfunction of AQP0 has a more severe impact on the embryonic and fetal nucleus than the adult nucleus of the lens, consistent with different stages of AQP0 expression.

**Figure 5:**
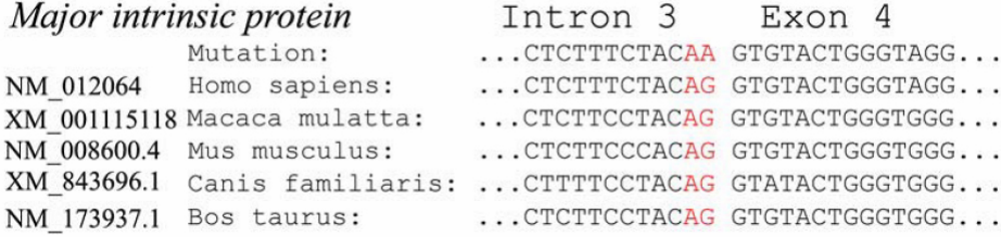
Multiple-sequence alignment of acceptor splice site in intron 3 of *MIP* from different species. It reveals that the canonical AG sites are conserved among different species.

Although several cases of splice sites with GT-TG, GT-CG, GC-AG, GG-AG, CT-AG, or AT-AC dinucleotides at the splice junctions were observed, the GT-AG rule is always obeyed [30]. It is reported that 87% of the 3′ splice-site mutations involved the invariant AG dinucleotide [31]. As for *MIP*, the canonical AG sites in intron 3 are conserved among different species (Figure 5). Splice-site mutations were reported to result in exon skipping, activation of cryptic splice sites, creation of a pseudo-exon within an intron, or intron retention, among which exon skipping is the most frequent outcome [32]. Mutations in acceptor splice sites can result either in use of the acceptor site of the next intron, with consequent loss of exon skipping, or in the utilization of a cryptic acceptor splice site upstream the mutation sites or in the next exon [33]. In our present study, the mutation occurred in the invariant AG dinucleotide of the last intron, so no existing canonical AG sites downstream are available to be used as the alternative acceptor splice site. Therefore, one or more cryptic acceptor splice sites are supposed to be used for aberrant splicing. According to the NNSPLICE program [34], we detected 8 possible cryptic acceptor splice sites scored more than 0.4 in intron 3 and exon 4. They were respectively located at +1599nt (0.81), +1758nt (0.52), +1850nt (0.52), +2588nt (0.64), +2735nt (0.92), +3040nt (0.44), +3074nt (0.88), and +3148nt (0.44) of *MIP*. Further study is required to confirm the above sites.

Several previous studies have demonstrated that the AQP0 COOH-terminus is very crucial to lens development and transparency through interactions with calmodulin, cytoskeletal proteins filensin and CP49, and connexin 45.6 [35-37]. Cleavage of the intracellular COOH-terminus decreases water permeability and enhances the adhesive properties of the extracellular surface of AQP0, indicating a conformational change in the molecule [38,39]. The possible aberrant splicing of *MIP* pre-mRNA may disrupt the normal COOH-terminus of AQP0, and leads to disbalance of the lens internal homeostasis, which is necessary to maintain transparency, and finally results in cataract formation. In addition, mutation analysis of AQP0 transcripts from the Cat^Fr^ lens indicated that the Cat^Fr^ mutation resulted in substitution of a long-terminal repeat sequence for the COOH-terminus of *Mip* (AQP0-LTR) [40]. AQP0-LTR in Cat^Fr^ was accumulated in sub-cellular compartments and made mature fiber cells fail to stratify into uniform, concentric growth, and finally resulted in congenital cataract [41,42].

In conclusion, we first describe the identification of an acceptor splice-site mutation in human genes (IVS3 −1 G>A in *MIP*) associated with ADCC, characterized by “snail-like” cataract phenotype. Further investigation is required to elucidate the pathogenesis of the novel splice-site mutation of the *MIP* gene on cataract formation.
